# Peroxiredoxin 2: An Important Element of the Antioxidant Defense of the Erythrocyte

**DOI:** 10.3390/antiox12051012

**Published:** 2023-04-27

**Authors:** Izabela Sadowska-Bartosz, Grzegorz Bartosz

**Affiliations:** 1 Laboratory of Analytical Biochemistry, Institute of Food Technology and Nutrition, College of Natural Sciences, University of Rzeszow, 4 Zelwerowicza St., 35-601 Rzeszow, Poland; 2 Department of Bioenergetics, Food Analysis and Microbiology, Institute of Food Technology and Nutrition, College of Natural Sciences, University of Rzeszów, 4 Zelwerowicza St., 35-601 Rzeszow, Poland; gbartosz@ur.edu.pl

**Keywords:** antioxidant, calpromotin, erythrocyte, hydrogen peroxide, glutathione, peroxiredoxin, thioredoxin

## Abstract

Peroxiredoxin 2 (Prdx2) is the third most abundant erythrocyte protein. It was known previously as calpromotin since its binding to the membrane stimulates the calcium-dependent potassium channel. Prdx2 is present mostly in cytosol in the form of non-covalent dimers but may associate into doughnut-like decamers and other oligomers. Prdx2 reacts rapidly with hydrogen peroxide (k > 10^7^ M^−1^ s^−1^). It is the main erythrocyte antioxidant that removes hydrogen peroxide formed endogenously by hemoglobin autoxidation. Prdx2 also reduces other peroxides including lipid, urate, amino acid, and protein hydroperoxides and peroxynitrite. Oxidized Prdx2 can be reduced at the expense of thioredoxin but also of other thiols, especially glutathione. Further reactions of Prdx2 with oxidants lead to hyperoxidation (formation of sulfinyl or sulfonyl derivatives of the peroxidative cysteine). The sulfinyl derivative can be reduced by sulfiredoxin. Circadian oscillations in the level of hyperoxidation of erythrocyte Prdx2 were reported. The protein can be subject to post-translational modifications; some of them, such as phosphorylation, nitration, and acetylation, increase its activity. Prdx2 can also act as a chaperone for hemoglobin and erythrocyte membrane proteins, especially during the maturation of erythrocyte precursors. The extent of Prdx2 oxidation is increased in various diseases and can be an index of oxidative stress.

## 1. Introduction

Erythrocytes are cells exposed continuously to oxidative stress due to their physiological function. They transport oxygen and contain hemoglobin (Hb) at a very high concentration. The oxygen-transporting function of Hb is not absolutely perfect; it is estimated that 1–3% of the erythrocyte oxyhemoglobin undergoes spontaneous heterolytic dissociation into methemoglobin (metHb) and superoxide radical anion O_2_^−●^ every day [1,2]. Enzymatic and spontaneous dismutation as well as reactions of O_2_^−●^ produce the more stable hydrogen peroxide (H_2_O_2_), so the erythrocyte is unavoidably exposed to a permanent flux of H_2_O_2_. Moreover, some drugs generate reactive oxygen species (ROS) and other radicals when interacting with Hb. Erythrocyte membranes contain phospholipids rich in polyunsaturated fatty acids, which are prone to peroxidation. Among others, the heme group of hemoglobin can initiate peroxidation of erythrocyte membrane phospholipids. Lipid peroxidation generates lipid peroxides and hydroperoxides. The hydroperoxides themselves are not very reactive but are readily converted in the presence of free iron and electron donor molecules to alkoxyl and hydroxyl radicals, which indiscriminately inflict damage on biomolecules. Intraerythrocyte parasites produce additional ROS [3,4]. Apart from ROS coming from inside the cells, erythrocytes may be exposed to ROS generated in tissues they contact. This exposure is augmented during intense exercise, at sites of inflammation, in ischemia/reperfusion and sepsis, and in various diseases such as neoplasia and diabetes [5,6,7,8]. Erythrocytes are often exposed to neutrophil-derived oxidants such as H_2_O_2_, peroxynitrite, hypochlorous acid/hypochlorite, and chloramines [9,10]. Moreover, external factors such as ionizing irradiation and xenobiotics, including environmental pollutants and heavy metals, also induce the formation of ROS [6,11,12].

**Figure 1:**
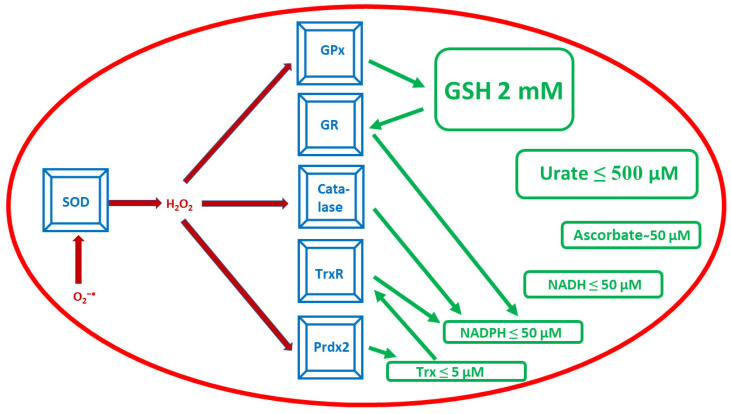
Main antioxidants (green) and antioxidant enzymes (blue) in the erythrocyte. Green lines indicate the interaction of enzymes with small-molecular-weight antioxidants (arrows point to the direction of oxidation). Glutathione peroxidase (GPx) uses GSH as a co-substrate; oxidized glutathione is regenerated by glutathione reductase (GR) at the expense of NADPH. Catalase binds and is protected by NADPH [31]. Peroxiredoxin 2 (Prdx2) uses thioredoxin (Trx) as a co-substrate. Oxidized Trx is reduced by thioredoxin reductase (TrxR) at the expense of NADPH.

What makes the situation even worse is that mammalian erythrocytes, as cells devoid of nucleus and protein synthesis machinery, are unable to synthesize new proteins to replace damaged ones during their lifespan (120 days for human erythrocytes). Thus, erythrocytes must be well equipped with an efficient antioxidant and chaperone machinery [13,14]. The main non-enzymatic erythrocyte antioxidant in concentration terms is glutathione (GSH), which is present at a concentration of about 2 mM [15,16], much higher than uric acid/urate (50–500 μM) [17,18], ascorbate (about 50 μM) [19,20,21], NADH (≤50 μM), NADPH (5–40 μM) [22,23,24], and thioredoxin (Trx) (3–5 μM) [25,26] (Figure 1). The primary role in the removal of ROS is played by antioxidant enzymes. CuZn-superoxide dismutase (SOD1) dismutates superoxide, while H_2_O_2_ was long believed to be disposed of only by catalase and glutathione peroxidase (GPx) [27,28]; it was debated which enzyme is more relevant for the decomposition of physiological levels of H_2_O_2_ [29,30]. This well-established picture was changed completely by the discovery of an antioxidant function of a relatively abundant erythrocyte protein.

It was long known that an increase in the intracellular free Ca^2+^ concentration in the erythrocyte up to micromolar levels activates a channel highly selective for potassium over other monovalent cations (the Gardos channel), resulting in a massive efflux of potassium from the cell [32]. A cytoplasmic erythrocyte protein calpromotin was found to be involved in the activation of the Gardos channel [33,34]. Later, it was proved that calpromotin, also known as a toroidal-shaped protein, single-torus protein, torin [35,36], or band 8 protein of the erythrocyte membrane [37], is identical to the natural killer enhancing factor-B (NKEF-B) [38], thiol-specific antioxidant protein (TSA) [39], thioredoxin-linked peroxidase [40], thioredoxin peroxidase B (TPxB), or peroxiredoxin 2 (Prdx2) [41].

An excellent review on the erythrocyte peroxiredoxin 2 was published by Christine Winterbourn and coworkers in 2008 [42]. The present article concentrates on (but is not limited to) data collected more recently. The articles were found in the PubMed and Google Scholar databases using the keywords “peroxiredoxin 2” and “erythrocyte” or “red blood cell”.

## 2. Peroxiredoxins

Peroxiredoxins (Prdxs; SH:H_2_O_2_ oxidoreductases, EC 1.11.1.15) constitute a large and highly conserved family of ubiquitous cysteine-dependent peroxidases that reduce hydrogen peroxide [41,43,44], organic peroxides [40,45], and peroxynitrite [46] at extremely high rates [47]. The active site of 2-Cys Prdxs is specifically arranged to perform a nucleophilic attack on the peroxyl O-O bond present in these compounds [41,48]. Oxidized Prdxs are regenerated to their reduced forms by other thiol compounds. These reactions are slower than the oxidation reactions but necessary to complete the catalytic cycle of Prdxs. Peroxiredoxins are abundant proteins that are almost always present in considerable excess over their substrates [41,48,49].

Based on the analysis of their active site-proximal sequences, Prdxs can be divided into six groups (Prdxs I-VI). All Prdxs possess the highly reactive cysteine (peroxidative cysteine, C_P_-SH), which forms a sulfenic acid (C_P_-SOH) in the reaction with peroxide. Depending on the catalytic mechanism of C_P_-SOH reduction, Prdxs can be divided into three subfamilies: (1) Typical 2-Cys Prdxs—in these proteins, C_P_-SOH reacts with a cysteine residue on another subunit of a constitutive non-covalent homodimer termed the ‘resolving’ cysteine (C_R_-SH); (2) atypical 2-Cys Prdxs—these proteins form disulfides involving C_R_-SH residing on the same subunit; and (3) 1-Cys Prdxs—in these proteins, C_R_-SH is absent, and other thiols are involved in the reaction with C_P_-SOH [50].

2-Cys Prdxs, where Prdx2 belongs, form obligatory non-covalent head-to-tail homodimers, which are minimal catalytic units [41,51]. The catalytic cycle of 2-Cys Prdxs consists of three main steps: peroxidation, resolution, and recycling. (i) In the peroxidation step, peroxide reacts with peroxidative cysteine C_P_ of one subunit of the dimer, oxidizing it to a sulfenic acid derivative (C_P_-SOH). (ii) A resolution step leads to the formation of disulfide bonds linking the C_P_ residue with the resolving cysteine C_R_ of another subunit. The formation of this disulfide bond is often accompanied by the dissociation of decamers into dimers [52,53]. (iii) Recycling consists of the regeneration of the Prdx into the reduced state by the thioredoxin/thioredoxin reductase (Trx/TrxR) system [49] or perhaps also by other thiol systems [54,55,56,57].

In contrast to prokaryotic 2-Cys Prdxs, the eukaryotic ones possess two architectural elements: an internal GGLG-containing loop and C-terminal YF motifs, which are well-spaced [41]. The interaction between these motifs requires transformation from a fully folded to a locally unfolded conformation. This reaction is relatively slow and therefore decreases the rate of disulfide bond formation. As a result, the C_P_-SOH can react with a second H_2_O_2_ molecule and becomes hyperoxidized to the cysteine sulfinic acid (C_P_-SO_2_H). Under conditions of extreme oxidative stress, this latter species can be further oxidized to cysteine sulfonic acid (C_P_-SO_3_H). This process, which is called hyperoxidation or overoxidation, causes the inactivation of the Prdx. The efficacy of hyperoxidation in various 2-Cys Prdxs is affected by the C-terminal amino acid sequence surrounding C_R_ [58].

Mammalian cells contain four isoforms of 2-Cys Prdxs: cytosolic Prdx1 and Prdx2, mitochondrial Prdx3, and Prdx4 localized in the endoplasmic reticulum. In typical 2-Cys Prdxs, the conserved C_R_ in the C-terminal region is unable to compete with C_P_-SH for peroxide but is able to form a disulfide bond with C_P_-SOH on the other subunit (resolve C_P_-SOH). The disulfide can be efficiently reduced by Trx, and oxidized Trx is regenerated by TrxR at the expense of NADPH oxidation [43,52]. Because of oxidizing Trx, 2-Cys Prdx enzymes are thus also called thioredoxin peroxidases.

AhpC, a bacterial Prdx, is extremely resistant to hyperoxidation; eukaryotic cytosolic Prdx2 is highly sensitive, while mitochondrial Prdx3 shows an intermediate sensitivity [56,59]. The sulfinic acid (C_P_-SO_2_H) but not the sulfonic acid (C_P_-SO_3_H) can be reduced by sulfiredoxins (Srxs) and possibly sestrins [60,61,62].

Sulfiredoxin was first identified in *Saccharomyces cerevisiae* as a product of a gene induced by H_2_O_2_ treatment [60]. Further analysis showed that Srx was able to reduce the hyperoxidized form of Prdx in a process dependent on the presence of ATP-Mg^2+^ and an exogenous reductant (dithiothreitol, Trx, or GSH). The reaction of sulfiredoxin is slow and involves several steps. First, Srx catalyzes the phosphorylation of C_P_-SO_2_H by ATP. Then, the sulfinic phosphoryl ester is hydrolyzed by Srx to form a thiosulfinate with Prdx. The next step involves a thiol, usually GSH, to produce thiolated Srx and C_P_-SOH. Further reduction steps are needed to bring sulfenylated Prdx to its reduced form as well as an additional thiol to recycle Srx [60,63].

Obviously, the energy cost of reduction of hyperoxidized Prdxs is high and suggests the significance of the process. Structural studies of hyperoxided Prdx2 showed that C_P_-SO_2_H is buried within the active site and therefore it was unclear how mechanistically Srx could get access to it. Crystal structure analysis of the Prdx-Srx complex revealed tightly intertwined proteins with the C-terminus of Prdx completely unfolded and packed on the backside of Srx away from the Srx active site [64].

Human Srx exhibits a ubiquitous tissue distribution, although the expression level varies greatly [63,65]. The k_cat_ values for the rat, human, and *Arabidopsis thaliana* Srx range from 0.1 to 1.8 min^−1^. Thus, Srx is an inefficient enzyme. It is thought that this low activity is of physiological relevance, as the Prdx molecules may require slow repair so that downstream, H_2_O_2_-mediated signaling events can be potentiated. In tissues with low intracellular activities of Srx and Trx/ThrR activities, irreversibly hyperoxidized Prdx2 may accumulate in the cells [65].

A remarkable feature of reduced dimers of 2-Cys Prdxs is their tendency to form high-molecular-weight complexes consisting (depending on the particular protein) of five or six dimers. The aggregation increases their reactivity with H_2_O_2_ [66]. The hyperoxidation of 2-Cys Prdxs can also lead to the formation of spherical aggregates of very high molecular mass (>2000 kDa) believed to play a molecular chaperone function that can prevent the unfolding and precipitation of model proteins [67,68].

Prdxs play three complementary roles in the cells: they (i) scavenge ROS, (ii) participate in ROS signaling, and (iii) act as molecular chaperones.

Given their high abundance within cells and high reactivity with H_2_O_2_, Prdxs seem ideally suited to participate in H_2_O_2_-mediated intracellular signaling [69]. Two models have been proposed to explain how hyperoxidation of the peroxidative cysteine functions in redox signaling. The “floodgate” model proposes that hyperoxidation of Prdxs allows for a local accumulation of H_2_O_2_ and activation of redox signaling pathways [41,70,71]. Oxidized Prdx can also oxidize other proteins; in this pathway, Prdxs act as sensors of H_2_O_2_ and pass oxidizing equivalents to other thiol proteins such as phosphatases and transcription factors via a relay mechanism [72]. Alternatively, the “smoke alarm” model proposes that oxidation of a Prdx alters its structure and oligomerization, affecting its ability to interact with other signaling proteins such as kinases, phosphatases, and transcription factors and modify their activities, alerting the cell of oxidative stress [73]. However, both these roles concern cells more complex than erythrocytes.

Another aspect of Prdx hyperoxidation is that it releases Trx from the duty of Prdx regeneration, allowing it to reduce other oxidized proteins [74,75].

Peroxiredoxins are abundant proteins. They are among the 10 most abundant proteins of *Escherichia coli* [76]. In mammalian cells, they constitute 0.1–0.8% of the soluble proteins [43]. Prdx2 is the most abundant in mammalian neurons, making it a prime candidate to defend against oxidative stress [77]. In blood, Prdx2 is found in erythrocytes, monocytes, and T and B lymphocytes but not in granulocytes. It is normally not present in blood plasma unless small amounts of the protein are released from cells [41,53,78].

## 3. Human Erythrocyte Peroxiredoxin 2

**Figure 2:**
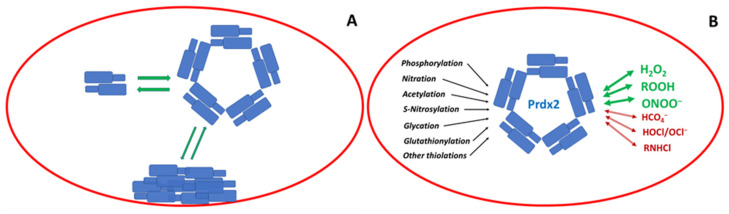
Prdx2 in erythrocyte: structure, substrates, and post-translational modifications (presented in Section 3, Section 4 and Section 5). Prdx2 is active as non-covalent dimers, which may form decamers and interact with the erythrocyte membrane (**A**). Prdx2 interacts with a range of substrates and under physiological conditions is the main enzyme removing some of them (indicated in green). Within the cell, it is subject to several post-translational modifications (**B**).

In the erythrocyte, Prdx2 is the third most abundant protein (Section 3.1). Its basic functional unit is a non-covalent dimer (Section 3.2), but dimers associate into decamers and possibly other oligomers (Section 3.3). A fraction of the protein is bound to the erythrocyte membrane (Section 3.4). Prdx2 reacts with hydrogen peroxide at an extremely high rate; oxidized Prdx2 is reduced by Trx or may be reduced by other thiols (Section 3.4). Apart from hydrogen peroxide, Prdx2 reacts efficiently with other hydroperoxides (Section 4.1 and Section 4.2) and peroxynitrite (Section 4.3). It reacts also with peroxymonocarbonate (Section 4.4) as well as hypochlorous acid/hypochlorite and chloramines (Section 4.5). Erythrocyte Prdx2 is subject to post-translational modifications such as phosphorylation (Section 5.1), nitration (Section 5.2), acetylation (Section 5.3), *S*-nitrosylation (Section 5.4), glycation (Section 5.5), glutathionylation (Section 5.6), and formation of other mixed thiols (Section 5.7) (Figure 2).

### 3.1. Abundance in Erythrocyte

The amount of Prdx2 in erythrocytes was estimated as 5.6 mg/mL of packed erythrocytes in the cytosol and 2.94 μg/mL packed erythrocytes (about 0.05%) as membrane-bound. Assuming a monomer molecular weight of 23,000, the concentration of calpromotin within the cytoplasm is 0.24 mM (per monomer), which corresponds to about 14 million copies per cell [34]. Thus, Prdx2 is the third most abundant protein in the erythrocyte after hemoglobin and carbonic anhydrase. More recently, an HPLC method of Prdx2 quantification showed average levels of Prdx2 in male and female subjects of 7.28 μg/mg and 8.29 μg/mg erythrocyte protein, respectively; the difference between the sexes was not statistically significant [79]. These values, which corresponded to about 2.4 mg Prdx2/mL of packed erythrocytes, were about two times lower than those reported previously [33,34]. However, the older values will be assumed in this review because they are used by most authors. Erythrocytes also contain Prdx1 (another 2-Cys Prdx) and Prdx6 (one-Cys Prdx) but in much lesser amounts than Prdx2 [80]. The accumulation of Ptdx2 is maximal in the early stages of erythrocyte development (prior to the appearance of hemoglobin) [81].

### 3.2. Structure of the Prdx2 Dimer

The human erythrocyte Prdx2 monomer is a 198-amino-acid protein. This protein migrates as a monomer in electrophoresis under reducing conditions, while under non-reducing conditions is almost completely dimerized, which indicates that it exists in the native state mostly as a non-covalent dimer of 22 kD subunits [14].

A crystallographic study of Prdx2 from human erythrocytes showed that the protein monomer consists of domain I (residues 2–169) and the C-terminal arm (residues 170–198). Domain I contains seven β sheets, five α helices, and two 3_10_ helices. A thioredoxin fold is defined by four of the seven β strands (β3, β4, β6, and β7) and four flanking α helices (α1, α2, α4, and α5), where α1 and α2 together form a single kinked helix. 

The ellipsoidal dimer (52 Å × 53 Å × 60 Å) is more compact in comparison to the monomer. The two monomers are related by a molecular dyad, and the C-terminal arm of one subunit folds over domain I of the other monomer. The β sheets of each subunit combine to form a 14-stranded β sheet fused between the respective seventh strands by five mainchain and sidechain hydrogen bonds involving Arg139, Gln140, Ile141, Val143, Asn144, and Asp145 as well as a hydrophobic interaction between Ile141 and Val143. The monomer–monomer interface is stabilized by a further 15 hydrogen bonds and 12 hydrophobic interactions between other residues.

The active-site Cys51 (peroxidative cysteine, C_P_) is positioned at the N terminus of helix α, which forms a βαβ substructure along with the closely associated helix α2 and the two adjacent parallel strands, β3, and β4. In the fully folded (FF) compact state, the active site contained in the α2 helix is buried inside a hydrophobic pocket that opens close to the dimer–dimer interface, while the resolving cysteine C_R_ (Cys172) is partially exposed. The active-site pocket around Cys51 is formed by the residues Tyr43, Pro44, Thr48, Val50, Glu54, Trp86, Arg127, and Arg150 and also by Val171 from the other subunit. The local unfolding of the α2 helix (LU state) is necessary to expose the C_P_ (in order for the resolving cysteine C_R_ from the other subunit of the dimer to react with C_P_ and form a disulfide bond). This localized unfolding brings the two cysteines, which in the fully folded (FF) state are separated by a distance of ~13 Å, into closer proximity [82,83]. Tyr43 and Val171 are conserved in 2-Cys mammalian Prdxs, while the other listed residues are conserved among all mammalian Prdxs [83]. 

### 3.3. Formation and Structure of Prdx2 Oligomers

In erythrocytes Prdx2 exists in solution as a dynamic equilibrium of dimers (44 kDa) and decamers (220 kDa), perhaps cycling between these two states [84]. The decamers are regular structures that have a doughnut-like shape; they were visualized via transmission electron microscopy as a toroidal protein consisting of 10 subunits (and hence named torin) [35,82].

The structure is a toroid-shaped decamer constructed from five dimers that has a point group symmetry of 52. The toroid has a maximal diameter of ~130 Å, an internal diameter of ~60 Å, and a thickness of ~50 Å. The interactions at the dimer–dimer interface are mainly hydrophobic with some water-mediated hydrogen bonding, burying 630 Å^2^ of solvent-accessible surface area per monomer. In the decameric Trx2, Phe81 from the adjacent dimer restricts access to the active site. This apolar environment coupled with the limited solvent access appears to preserve the thiol status of Cys172 prior to its involvement in the catalytic cycle [82,83,84].

The dynamic equilibrium between the oligomers is known to be affected by various factors including protein concentration and its redox state, pH, ionic strength, and Ca^2+^ concentration. The formation of cytosolic decamers of Prdx2 in vivo seems to be promoted by a small decrease in pH [39], changes in the salt concentration [85], and changes in the Prdx2 oxidation state. Seemingly, decamers are formed by fully reduced dimers, and the reduced decamer is believed to be the most active form of Prdx2. The formation of disulfide bonds between monomers decreases the stability of the decamers. The formation of an inter-subunit disulfide bond in one or two dimers within the decamer is sufficient to induce complete decamer dissociation into dimers [82,86]. Hyperoxidation of Cys51 allows the formation of a stable decamer, which can be further stabilized by a salt bridge between the sulfinic acid group of Cys51 and Arg127 [41]. Other studies confirmed that the reduced or hyperoxidized forms of Prdx2 favor the decameric state, whereas the disulfide-bonded forms exist mainly as dimers [49,68,87,88], though not all reports are in full agreement with this conclusion.

Interestingly, Prdx2 isolated from human erythrocytes by Manta et al. [46] was an oxidized decamer. Prdx2 obtained from RBC lysates rapidly prepared after blood collection and immediately separated by using HPLC/size exclusion chromatography showed that the Prdx2 in human erythrocytes was present as oligomeric rather than monomeric or dimeric forms; there was little evidence that the oligomeric forms of native Prdx2 included overoxidized forms. Considering the decamer/dimer dissociation constant K_d_ < 10^−23^ M^4^, it should be expected that Prdx2 exists mainly as a decamer in vivo [46,89]. 

In vitro, the formation of a dodecahedron structure of human Prdx2 consisting of 12 decamers was reported, although the existence of such a structure in vivo is questionable [90]. Hyperoxidized Prdx2 in its high-molecular-mass structure was found to interact non-covalently with the protein disulfide isomerase ERp46 [54]. 

Prdxs are believed to harbor other proteins to protect them from inactivation and aggregation as molecular chaperones [41,67,68] (6.7). The presence of higher-molecular-weight oligomers (260–450 kDa) was found; they were reported to have a chaperone activity that was minimally enhanced by hyperoxidation induced by H_2_O_2_ treatment of erythrocytes [90]. Rinalducci et al. detected four Prdx2-containing protein heterooligomers in human erythrocytes. The highest-molecular-weight complex (440 kDa) contained decameric Prdx2 and tetrameric catalase (232 kDa). This complex contained both reduced and disulfide-linked dimers. Other complexes (of molecular weights 140, 100, and 67 kDa) contained only monomer subunits of Prdx2 (apparently seven, five, and three monomers) [91].

### 3.4. Binding of Prdx2 to the Erythrocyte Membrane

Oligomers of Prdx2 associated with the plasma membrane have been detected by many authors [33,34,35,36] and extracted from erythrocyte ghosts [33,36,92]. A significant correlation was found between the level of calpromotin (Prdx2) bound to the membrane and the potassium efflux; binding of less than 1% of the protein initiated the potassium efflux. Calcium was found to be required for the membrane binding of Prdx2. The membrane-bound fraction of Prdx2 was increased by leupeptin, a stimulator of the transport, and diminished by iodoacetic acid, an inhibitor of calcium-activated potassium transport [33,34].

The fraction of erythrocyte membrane-associated Prdx2 increases under oxidative stress [93,94,95,96] as well as hypoxia [97,98]. Rocha et al. found no membrane-associated Prdx2 in intact erythrocytes but observed that treatment of erythrocytes with hydrogen peroxide in the presence of azide (to inhibit catalase) induced Prdx2 binding to the membrane; the effect was augmented with an increasing H_2_O_2_ concentration [93]. However, studies with purified Prdx2 and isolated ghosts showed that the interaction was independent of the Prdx2 redox state [99]. Contrary to these results, Bayer et al. found a decrease in Prdx2 binding to the erythrocyte membrane with increasing concentrations of H_2_O_2_ [100]. Sharma et al. found membrane-bound Prdx2 in untreated erythrocytes but observed that heat treatment increased the amount of membrane-bound Prdx2 [101].

The mode of membrane association of Prdx2 is not clear. Prdx2 was found to bind to integral membrane proteins of cell membranes via its C-terminal region (Asp-187-Gln-197) [40,92]. Band 3 was identified as one of the membrane binding sites of Prdx2 (and perhaps the main). Prdx2 interacts electrostatically with the 55-amino acid N-terminal domain of the Band 3 protein; in particular, the first 11 amino acids of the N-terminal peptide have a pronounced acidic character and could be considered a good candidate for binding to the center of the ring of decameric Prdx2. Prdx2 did not bind to the membranes of erythrocytes of a Band 3 Neapolis patient; a truncated isoform of the Band 3 protein lacked the N-terminal 11 amino acid residue. Interaction between recombinant Prdx2 and the cytoplasmic N-terminal domain of the Band 3 protein is characterized by a K_d_ of 34 nM, indicating a high-affinity interaction. The stoichiometry of the interaction between the cytoplasmic terminal domain of the Band 3 protein and Prdx2 is one dimer of the cytoplasmic Band 3 domain per five dimers of Prdx2. The binding of Prdx2 to the Band 3 protein induces small conformational changes in Prdx2 [95].

The Band 3 protein of the erythrocyte membrane is also the site of interaction of deoxyHb, metHb, and hemichromes with the membrane, and competition for the common binding site may affect the membrane association of Prdx2. DeoxyHb binds a synthetic peptide of the cytoplasmic domain of the Band 3 protein containing the first 11 amino acid residues with a K_d_ of 0.3 mM at pH 7.2 [102], a value 10^5^ times lower than that for the binding of Prdx2. Increased membrane association of Prdx2 upon deoxygenation suggests that Prdx2 and deoxygenated hemoglobin do not share a common binding site. However, other data speak in favor of the competition of Prdx2 with hemoglobin derivatives for the same binding sites on the membrane. Inhibition of Prdx2 by conoidin A leads to an increase in its membrane linkage and to a decrease in membrane-bound hemoglobin, suggesting in turn that they share the same membrane-binding site [103]. A decrease in Prdx2 binding to the erythrocyte membrane under oxidative stress conditions was also observed in erythrocytes of beta-thalassemic mice, and ascribed to the increased metHb binding to the membrane, reducing the access of Prdx2 to the same common binding site [94]. Hemichromes are expected to bind to the Band 3 protein with a much higher affinity than hemoglobin [101,104]. Prdx2 did not bind to the membrane of intact erythrocytes treated with phenylhydrazine (PHZ) containing membrane-bound hemichromes, but it did bind to erythrocyte membranes that were oxidized with PHZ in the absence of hemoglobin and contained no hemichromes [95]. 

The P rdx2 decamer has no positively charged surface areas and is therefore unlikely to interact directly with phospholipid head groups in the membrane. There are hydrophobic patches on the outer rim of the torus and acidic and hydrophobic patches on the torus face that could enable interaction with membrane-bound proteins such as stomatin (Band 7.2b) [92]. Indeed, Prdx2 was found to bind to lipid vesicles. Upon binding to phosphatidylserine or phosphatidylglycerol, dimeric human Prdx2 is assembled into trefoil-shaped small oligomers (possibly hexamers) with full chaperone and null peroxidase activities. Spherical high-molecular-weight complexes were formed only when phosphatidylserine or phosphatidylglycerol was bound to overoxidized Prdx2. Thus, these lipids with a net negative charge, which can be supplied by increased membrane trafficking under oxidative stress, may induce a structural and functional switch of Prdx2 [105].

It has been speculated that the membrane-bound Prdx2 may be involved in the protection against membrane lipid peroxidation [40,106].

### 3.5. Reaction Kinetics of Human Erythrocyte Prdx2

A key feature of 2-Cys Prdxs is the high reactivity of their reduced peroxidative cysteine residue C_P_ with H_2_O_2_ (with rate constants in the range of 10^7^–10^8^ M^−1^ s^−1^) [107]. Originally, a second-order rate constant for the reaction of Prdx2 with H_2_O_2_ of approximately 10^5^ M^−1^ s^−1^ was found [45]. However, much higher values were subsequently obtained on the basis of competition with horseradish peroxidase or catalase (2–4) × 10^7^ M^−1^ s^−1^ [107,108,109]. The measurements based on the loss of intrinsic fluorescence of Prdx upon oxidation by H_2_O_2_ resulted in even higher values of around 10^8^ M^−1^ s^−1^ [110]. The latter values were more than a million times faster than those for the reactions of H_2_O_2_ with low-molecular-weight thiols [111]. Such enzymatic efficacy allows Prdxs to not only join the list of the primary antioxidants but places them among the fastest enzymes (if considering the reaction with H_2_O_2_) [56]. For comparison, second-order rate constants for the reactions with H_2_O_2_ were reported to be 3.4 × 10^7^ M^−1^ s^−1^ for catalase [112] and 2.1 × 10^7^ M^−1^ s^−1^ for glutathione peroxidase [113].

The reactivity of cysteine residues with hydrogen peroxide depends on their ionization state [111]. The C_P_-SH of 2-Cys Prdxs has a pK_a_ value of about 6 [114], which makes it mostly ionized at physiological pH. This low pK_a_ of the active site thiol is insufficient to confer the high peroxide reactivity. To obtain a sufficient rate enhancement, it is necessary to activate the peroxide. This involves the formation of a transition state in which there is hydrogen bonding of the peroxide to conserved arginine and threonine residues and amide nitrogen in the active site. Their concerted action stabilizes the transition state with the bound substrate, weakens the -O–O- bond, and brings the proximal O closer to C_P_-SH [49,66,71]. Apparently, even amino acids located further from C_P_-SH are involved in sustaining its high reactivity as their mutations sharply decrease the reaction rate [115]. Disruption of the Prdx2 structure in the region of the resolving cysteine C_R_ affects the reactivity of the peroxidative cysteine C_P_ with H_2_O_2_. Substituting Cys172 located near the C terminus and outside the active site with Ser (C172S mutation), which is expected to be minimally disruptive, does not affect the rates of oxidation of the reduced form and hyperoxidation of the sulfenic acid of C_p_. In contrast, the C172D and C172W mutations, which are expected to be more disruptive, decrease the rates of oxidation and hyperoxidation approximately 100-fold compared with WT. Thus, the C_P_ and C_R_ regions cannot be considered as structurally independent [116].

Studies by Peskin et al. demonstrated an intradimer cooperativity in Prdx2. The two peroxidative cysteines in both monomers of the dimer initially have identical reactivity with H_2_O_2_. However, the conversion of one C_P_ thiol to a sulfenic acid increases the rate of oxidation of the C_P_ in the second monomer by about 2.3-fold. In, contrast if the first site has time to condense to the disulfide before the second site is oxidized, then both sites react at the same initial rate. A negative cooperativity for the condensation of the sulfenic acid to disulfide was also identified. In this case, the conversion of one sulfenic acid in C_P_-SOH to a disulfide slows the rate of condensation of the second C_P_ to about 40% of the control value. Thus, the active sites within the Prdx2 dimer do not behave independently, and modification of one active site of Prdx2 influences the conformation of the other. As a consequence of the positive cooperativity for the sulfenic acid formation and negative cooperativity for condensation, the lifetime of the C_P_-SS-C_R_-C_P_-SOH form of the dimer in erythrocytes, cells with a low capacity for Prdx disulfide reduction is increased. Thus, the sulfenic acid in C_P_-SOH-C_P_-SS-C_R_ can have more time to condense before the disulfide is reduced, and negative cooperativity in the condensation decreases the rate of H_2_O_2_ removal under oxidative stress [117].

The reaction of Prdx2 with H_2_O_2_ is very fast, but the next steps of the catalytic cycle of Prdx2 are slower. Thus, although Prdxs are classified as enzymatic proteins, their reaction with H_2_O_2_ is in fact stoichiometric and not catalytic. 

The C_P_-SOH residue formed upon reaction with H_2_O_2_ can form a covalent dimer via disulfide with C_R_ of another monomer or be further oxidized (hyperoxidized) by the next H_2_O_2_ molecule to a sulfinyl derivative C_P_-SO_2_H. These reactions are characterized by the respective rate constants k_d_ and k_h_:

Rate of disulfide formation = k_d_ [Prdx2-C_P_-OH]

Rate of hyperoxidation = k_h_ [Prdx2-C_P_-SOH][H_2_O_2_]

The values of k_d_ and k_h_ were estimated to be about 2 s^−1^ and 10^4^ M^−1^ s^−1^, respectively. Thus, the sulfenic acid residue was oxidized 10^3^–10^4^ times more slowly than the thiolate but was nevertheless several orders of magnitude more reactive than most reduced thiol compounds [59].

The ratio of these rate constants and the concentration of hydrogen peroxide determine the rate of progressive inactivation by hyperoxidation during Prdx2 turnover. This rate was examined by several groups. One group found 1% inactivation at each cycle of disulfide formation [118]. Other reports indicated an even lower inactivation rate [109,116].

Reduction of Prdx2 by Trx was characterized by a second-order rate constant of 2.1 × 10^5^ M^−1^ s^−1^ [46]. The H_2_O_2_:thioredoxin peroxidase activity of human erythrocyte Prdx2 was characterized by K_m_ of 2.4 μM [46] (human thioredoxin) and 2.7 μM (rat thioredoxin) [43].

Prdx2 regeneration is especially slow. Therefore, high concentrations of Prdxs are required to quickly remove H_2_O_2_ and wait for reduction to the initial state, restoring its ability to react with the next H_2_O_2_ molecules. In a way, they can be compared to pipette tip racks: filled racks enable quick pipetting (removal of H_2_O_2_), especially with a multipette, while rack re-filling (regeneration) requires quite a time.

**Figure 3:**
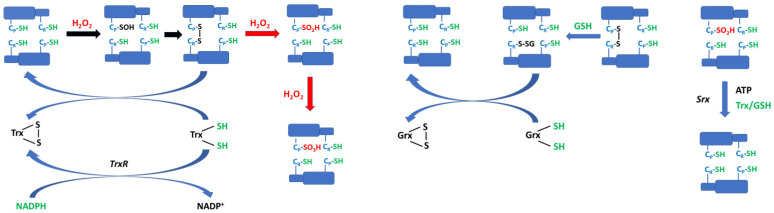
Reactions of Prdx2 with H_2_O_2_ can cause oxidation (black arrow) or hyperoxidation (red arrow). Oxidized Prdx2 can be reduced by thioredoxin (Trx) or glutathione (GSH) and glutaredoxin (Grx). Oxidized Trx is reduced by thioredoxin reductase (TrxR). Sulphinylated Prdx2 can be reduced by sulphiredoxin (Srx). For simplicity, oxidation and hyperoxidation of only one cysteine in each dimer is presented.

Instead of the Trx system, recycling may be performed by using GSH and glutaredoxin. C_P_-SOH and C_P_-SS-C_R_ can also react with GSH to form a mixed disulfide that is reduced by glutaredoxin [55,56]. These two systems can complement each other; e.g., in red blood cells, TrxR activity is low [14], and GSH/glutaredoxin could bear the burden of Prdx recycling of the Trx system [56] (Figure 3).

## 4. Substrates of Prdx2

The position of C_P_-SH in the active site allows an aggressive nucleophilic attack on the wide range of substrates of the ROOH type. Apart from hydrogen peroxide, Prdx2 reacts with lipid hydroperoxides and amino acid, protein, and other hydroperoxides as well as peroxynitrite, showing no exceptional reactivity with other thiol reagents such as iodoacetamide or chloramines [56,108].

### 4.1. Lipid Hydroperoxides

Prdx2 exhibits high catalytic activity for the reduction of the fatty acid hydroperoxides as evidenced by K_m_ and V_max_ values of 89.9 μM and 28.64 μmol^−1^ min^−1^ mg^−1^, respectively, for linoleic acid hydroperoxide. Taking into account the ability of Prdx2 with model phospholipids, it was postulated that Prdx2 reduces hydroperoxides in the erythrocyte membrane [40].

There are, however, considerable differences between the reactivities of H_2_O_2_ and organic hydroperoxides toward Prdx2 when whole human erythrocytes are treated. Insignificant hyperoxidation of Cys51 of Prdx2 was observed at low doses of H_2_O_2_. The extent of covalent dimer formation in the presence of *tert*-butyl hydroperoxide was lower than that in the presence of H_2_O_2_, but substantial monomer and dimer hyperoxidation occurred up to sulfinic and sulfonic derivatives, unlike after hydrogen peroxide treatment [47,119]. Arachidonic acid lipid hydroperoxide metabolites of 5-, 12-, 15-lipoxygenase-1, and cyclooxygenase-2 such as 5-, 12-, and 15-hydroperoxy eicosatetraenoic acids also hyperoxidized Prdx2 [120]. Hyperoxidized Prdx2 was suggested to be a potential marker of oxidative injury caused by organic hydroperoxides in human erythrocytes [119]. As GPxs are not inactivated by organic hydroperoxides and reduce them efficiently [121], GPx1 and GPx4 (phospholipid hydroperoxide glutathione peroxidase) [122,123] rather than Prdx2 appear to play the primary role in detoxifying organic peroxides in the erythrocyte.

### 4.2. Other Hydroperoxides

Uric acid, the end product of purine metabolism in humans, is present in the blood in plasma in concentrations ranging from 50 to 500 µM in healthy individuals mainly in the dissociated form as urate (pK_a_ of uric acid is 5.4). Urate can be oxidized by myeloperoxidase and lactoperoxidase to generate urate free radical and urate hydroperoxide [124,125]. Urate hydroperoxide oxidizes methionine and cysteine and reacts with glutathione at a rate constant of 13.8 M^−1^ s^−1^ [126]. Urate hydroperoxide was found to be a good substrate of Prdx2; the second-order rate constant for the reaction was 2.3 × 10^6^ M^−1^ s^−1^, making Prdx2 the main reactant for this compound in the erythrocyte. Oxidation of Prdx2 by urate hydroperoxide might affect cell function and be partially responsible for the pro-oxidant and pro-inflammatory effects of uric acid [110].

Amino acid and especially protein peroxides are among the main products formed by oxidants in the cells [127]. Although the rate constants for the amino acid hydroperoxides with Prdx2 are not as high as those for H_2_O_2_, they have approached values measured for small alkyl hydroperoxides. The second-order rate constants for the reaction of Prdx2 with lysozyme hydroperoxide, bovine serum albumin hydroperoxide, histidine hydroperoxide, and N-acetyl leucine hydroperoxide were found to be 40 × 10^3^, 160 × 10^3^, 2 × 10^3^, and 4 × 10^4^ M^−1^ s^−1^, respectively. In erythrocytes and erythrocyte lysates, oxidation of Prdx2 by amino acid and lysozyme peroxides, respectively, proceeded faster than the oxidation of GSH. Prdxs are the only thiol proteins shown to react efficiently with protein hydroperoxides. Prdx2 is oxidized by the hydroperoxides to disulfide-bonded dimers, which can be reduced by Trx. Thus, this process can be considered a catalytic mechanism for breaking down amino acid and protein hydroperoxides. However, if the recycling is slow, it is perhaps more realistic to treat Prdx2 as a scavenger of the hydroperoxides that can be recycled [128].

### 4.3. Peroxynitrite

Peroxynitrite, a potent oxidant formed by the diffusion-controlled reaction between O_2_^−●^ and nitric oxide (NO^●^), can easily permeate the erythrocyte membrane. During an inflammatory process, the activation of phagocytic cells induces the expression of iNOS, producing high levels of NO^●^. Nitric oxide reacts with O_2_^−●^ formed in the respiratory burst to form peroxynitrite. Even though intravascularly formed peroxynitrite can react with plasma components, an important fraction diffuses into RBC [10,129]. The second-order rate constant for the reaction of Prdx2 with peroxynitrite (1.4 × 10^7^ M^−1^ s^−1^) at pH 7.4 is similar to that for the reaction with hydrogen peroxide. It was estimated that ca. 27% of the peroxynitrite in the bloodstream is cleared by circulating erythrocytes, mainly by Prdx2. Peroxynitrite hyperoxidizes Prdx2 as well [46].

### 4.4. Peroxymonocarbonate

The rate of hyperoxidation of Prdx2 in situ may be higher than predicted based on studies of model systems due to the presence of the peroxymonocarbonate anion HCO_4_^−●^. Bicarbonate buffers are seldom used in in vitro experiments, while bicarbonate is present in erythrocytes, which play a crucial role in its transport and removal. The peroxymonocarbonate anion is formed in a reaction between hydrogen peroxide and carbon dioxide/bicarbonate [130,131]:

H_2_O_2_ + CO_2_ ⇆ HCO_4_^−●^ + H^+^

H_2_O_2_ + HCO_3_^−^ ⇆ HCO_4_^−●^ + H_2_O

HCO_4_^−●^ is a more reactive oxidant than H_2_O_2_; it reacts with low-molecular-weight thiols and methionine about 100 times faster than H_2_O_2_ and can be expected to react fast with Prdx2. However, only about 1% of H_2_O_2_ is present as HCO_4_^−●^ at physiological bicarbonate concentrations at pH 7, so the rate of enhancement of the reaction for thiols such as GSH is only about 2-fold [130,132]. The presence of the small fraction of H_2_O_2_ in the form of HCO_4_^−●^ has no detectable effect on the measured initial oxidation step of Prdx2 but 25 mM bicarbonate/CO_2_ increased the ratio of hyperoxidation of Prdx2 6-fold [133].

### 4.5. HOCl and Chloramines

Hypochlorous acid (HOCl) is formed mainly by the oxidation of chloride ions by H_2_O_2_ in a reaction catalyzed by myeloperoxidase. At physiological pH, HOCl exists in equilibrium with its anion HOCl (the pK_a_ of HCl dissociation is 7.6 [134]). HOCl/OCl^−^ can react with amines to produce chloramines, which are less reactive than hypochlorite but retain reactivity for longer. Erythrocyte Prdx2 is oxidized by HOCl/OCl^−^ and cell-permeable chloramines, but its reactivity is comparable to that of GSH, so Prdx2 is not the main target for these compounds. The second-order rate constants for the reactions of NH_2_Cl with GSH and Prdx2 are 1 × 10^3^ M^−1^ s^−1^ and 1.5 × 10^4^ M^−1^ s^−1^, respectively, for the reactions of glycine chloramine 230 M^−1^ s^−1^ and 8 M^−1^ s^−1^, respectively, and for reactions of taurine chloramine 120 M^−1^ s^−1^ and 3 M^−1^ s^−1^, respectively [135].

## 5. Post-Translational Modifications of Prdx2

Like any other protein, erythrocyte Prdx2 can undergo covalent modifications that affect its activity.

### 5.1. Phosphorylation

Several threonine/serine residues [136] and two tyrosine residues [137] can be phosphorylated in the Prdx2 molecule. Changes in the phosphorylation of Prdxs might affect not only their activity but also their molecular size by shifting the dimer–decamer equilibrium. Phosphorylation of Prdx2 at Thr89 by Cdk5 kinase resulted in a diminution of the peroxidase activity of the protein [136]. In vitro studies of recombinant Prdx2 identified two candidate tyrosine kinases capable of phosphorylating Prdx2: Syk phosphorylating Tyr193 and Fyn phosphorylating Tyr115 residues on Prdx2. The amount of Prdx2 phosphorylated by the Syk kinase in vitro is higher than that phosphorylated by the Fyn kinase. Phosphorylation of Tyr193 elevates Prdx2 activity. Tyr 115 is located in a hydrophobic cleft remote from the active site, and its modifications should affect the protein function less. On the other hand, Tyr193 is situated in a key region both for the catalytic activity and for the control of the oligomeric structure, so its phosphorylation may have a stronger effect on the activity of Prdx2. Bioinformatic analysis suggests that phosphorylation of Tyr193 favors the transition from the FF to the LU conformation, promoting disulfide formation and inhibiting C_P_ hyperoxidation [137].

### 5.2. Nitration

Erythrocyte Prdx2 not only reacts with peroxynitrite but can also be nitrated during catalysis [46]. The mechanism of tyrosine oxidation begins with the formation of the tyrosyl radical followed by the addition of nitrogen dioxide to yield 3-nitrotyrosine. One of the major pathways of protein nitration in vivo involves the reaction of radicals derived from peroxynitrite homolysis [10]. Tyrosine nitration increases both Prdx2 peroxidase activity and the resistance to hyperoxidation by H_2_O_2_ [138].

Treatment of oxidized (C_P_-SS-C_R_) Prdx2 with a 5-fold excess of peroxynitrite produced mainly mononitrated and dinitrated species. Nitration of three residues (Tyr33, Tyr126, and Tyr193) was confirmed [138]; nitration of Tyr193 was necessary to produce an increase in Prdx2 activity and its resistance to hyperoxidation [138,139]. Prdx2 was phosphorylated by the Syk kinase in red cells exposed to oxidation induced by diamide and was a specific target of Syk [139]. 

### 5.3. Acetylation

N-acetylation of Lys196 in Prdx2 causes an increase in peroxidase activity, a decrease in hyperoxidation susceptibility, and an elevation of its resistance to transition to high-molecular-mass complexes. Prdx2 belongs to specific targets of histone deacetylase HDAC6. The acetylated form of Prdx2 accumulates in the absence of an active HDAC6 [140].

### 5.4. S-Nitrosylation

Prdx2 is *S*-nitrosylated (forming cysteine-NO derivatives) by reaction with nitric oxide at both critical cysteine residues (C_P_ and C_R_). Nitrosylation was found to promote disulfide formation between C_P_ and C_R_, disrupt the oligomeric structure of Prdx1, and prevent the reaction with peroxides as well as regeneration by Trx [77,141].

### 5.5. Glycation

Prdx2 becomes glycated (mostly on Arg138 and Lys195); the extent of glycation increases with increasing glycemic index [142].

### 5.6. Glutathionylation

Prdx2 readily forms glutathionylated products on one or both of its active site C_P_ and C_R_ residues when oxidized in the presence of physiological concentrations of GSH. Peskin et al. reported reaction rate constants allowing for estimation of the possibility of Prdx2 glutathionylation under in vivo conditions (Table 1). They were obtained for a C172S mutant to exclude the resolving cysteine from the reactions.

Two mechanisms of glutathionylation were identified, one involving disulfide exchange with oxidized Prdx2 and the other involving sulfenic acid. As a result of glutathionylation, inter-subunit disulfide formation is either reversed or inhibited, and hyperoxidation caused by modest excesses of H_2_O_2_ is suppressed. Thus, GSH could play a physiological role in protecting Prdx2 from hyperoxidation during oxidative stress. Most of the observed products were diglutathionylated on C_P_ and C_R_. C_R_ reacts slowly with H_2_O_2_, so it is unlikely to become glutathionylated via an oxidative mechanism. However, exchange between glutathionylated C_P_ and C_R_ can generate reduced C_P_, which can be rapidly oxidized to the sulfenic acid and then form a second GSH adduct. Thus, glutathionylation of C_P_ and C_R_ can be explained by a combination of oxidative and exchange mechanisms [55]. 

The GSH adducts were deglutathionylated efficiently by glutaredoxin 1 (Grx1), and GSH/Grx1 was able to support peroxidase activity. Thus, the GSH/Grx system should be considered as an alternative Prdx recycling mechanism (Figure 3). The Prdx recycling by GSH/Grx1 is apparently slower than by Trx/TrxR1. However, it may be more important when such TrxR activity is low (and this is the case for the erythrocyte). There may be more than one way of bypassing TrxR since Grx1 can also reduce Trx. Kinetic data with isolated Prdx2 imply that glutathionylation should be fast enough to occur in cells but if deglutathionylation by Grx1 is faster, glutathione adducts may not accumulate in cells containing active Grx1 [55].

Although Grx accounts for most of the deglutathionylation activity in mammalian cells, other enzymes may also participate in deglutathionylation of specific glutathionylated proteins under physiological conditions, one of them being sulfiredoxin [143].

### 5.7. Formation of Other Mixed Thiols

A similar mechanism of mixed disulfide formation as described above for GSH can operate for Prdx2 and other thiols (including protein thiols). Formation and resolution of a mixed disulfide would transfer oxidizing equivalents from the Prdx to the target protein and provide a thiol relay mechanism for signal transmission [55]. Such an interaction via a thiol relay mechanism was revealed for collapsin response mediator protein 2 (CRMP2), a protein ubiquitously expressed and that regulates the assembly and disassembly of microtubules in Jurkat T-lymphoma cells [144].

## 6. Inhibitors of Prdx2

Adenanthin, a diterpenoid isolated from the leaves of *Rabdosia adenantha*, a promising therapeutic agent for acute promyelocytic leukemia, binds to and inactivates Prdx2 [145]. C_P_ is approximately six times more reactive with adenanthin than C_R_. However, this inhibitor is not specific for Prdx2 and reacts also with other thiols. The rate constants for the reactions of adenanthin with the C_P_ and C_R_ of Prdx2 were 30 and 5 M^−1^ s^−1^, respectively; for the reaction with GSH, 20 M^−1^ s^−1^; and for the reaction with thioredoxin reductase, 1200 M^−1^ s^−1^, so other thiol compounds rather than Prdx2 are expected to be the main targets for adenanthin in erythrocytes and other cells [146]. 

**Figure 4:**
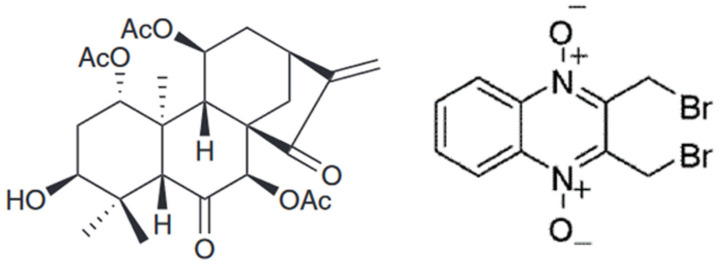
Structure of adenanthin (**left**) and conoidin A (**right**).

Conoidin A (2,3-bis(bromomethyl)-1,4-dioxide-quinoxaline), an inhibitor of host cell invasion by the human pathogen *Toxoplasma gondii* (Figure 4), inactivates Prdx2 by covalently binding to the C_P_ of the enzyme (IC_50_ = 23 μM) [147,148].

## 7. Status and Roles of Prdx 2 in the Erythrocyte

### 7.1. Oxidation Status In Situ

Determination of the extent of oxidation of Prdx2 in intact erythrocytes is not a trivial task since it is highly sensitive to H_2_O_2_. There may be sufficient amounts of H_2_O_2_ present in the buffer to cause complete oxidation of diluted protein. It was demonstrated that such mechanical factors as heating and mechanical shaking can produce nanomolar amounts of hydrogen peroxide in aerated aqueous solutions [149,150]. Phosphate buffers may contain low concentrations of H_2_O_2_ exceeding 50 nM [151]. The inclusion of low amounts of catalase in the buffers used for the preparation of Prdx2 or erythrocyte extracts prevented, though not completely, this oxidation. If catalase was included in the isolation buffer, Prdx2 was found to be practically completely reduced in fresh intact erythrocytes [14]. Another study reported that Prdx2 in erythrocytes without neutrophils was predominantly in the reduced form, with 6 ± 2% detected as the disulfide-linked dimer via non-reducing SDS-PAGE and Western blotting [152]. 

Monitoring of Prdx oxidation has proved valuable for measuring oxidative stress in erythrocytes. Exposure of erythrocytes to low micromolar concentrations of hydrogen peroxide results in immediate oxidation of Prdx2. It then takes at least 10 min for Prdx 2 to be converted back to its reduced form due to the low thioredoxin reductase activity in erythrocytes (0.034 nmol/10^6^ cells per min) [14,108]. In the meantime, Prdx2 can play no role in reducing hydrogen peroxide until regeneration. Thus, the presence or absence of Prdx2 may not affect the response of erythrocytes to higher doses of exogenous H_2_O_2_. In contrast, the rate of endogenous production of hydrogen peroxide is so low that the rate of formation of the Prdx2 dimer and its reduction allows for keeping whole or almost whole Prdx2 in the reduced state.

This low reduction capacity of oxidized Prdx2 is a specific feature of erythrocytes and some other cells (e.g., hepatocytes), while the majority of tumor cell lines maintain the capacity for the reduction of oxidized Prdx2 in slight excess over the maximal steady-state Prdx2 oxidation [153]. The use of 2,4-dinitrobenzene to inhibit the thiol regeneration systems that are present in isolated erythrocytes resulted in the accumulation of oxidized Prdx 2 without the addition of exogenous oxidants, confirming that Hb autoxidation is the major source of endogenous hydrogen peroxide responsible for Prdx2 oxidation. In line with this conclusion, bubbling of erythrocyte suspensions with CO to convert oxyhemoglobin to carboxyhemoglobin inhibited the increase in Prdx2 oxidation by approximately 65%. The high abundance in erythrocytes enables Prdx2 to handle up to an equivalent amount of H_2_O_2_ without the need for recycling [14].

Co-culture of erythrocytes with activated neutrophils resulted in the accumulation of oxidized Prdx2. No significant Prx2 oxidation was observed when erythrocytes were incubated with non-stimulated neutrophils. However, even at the low ratio of 1 stimulated neutrophil per 2000 erythrocytes, 23 ± 4% of the Prdx2 was oxidized, and at a ratio of 1:20, it was all in the form of covalent dimers. Prdx2 oxidation occurred rapidly. Dimerization was maximal at 5–15 min then gradually declined over the next 40 min. Phagocytosis of bacteria by neutrophils increased oxidation of erythrocyte Prdx2, although during phagocytosis hydrogen peroxide generation was localized to intracellular phagosomes. HOCl or other myeloperoxidase products made little contribution to Prdx2 oxidation. An increase in the Prdx2 oxidation was also observed in vivo when mice were injected with LPS and did not occur in mice lacking nitric oxide synthase 2. Oxidized Prdx 2 was also detected in erythrocytes isolated from the peripheral circulation of mice exposed to endotoxin [152]. Lowering the pH but not a temperature increase (up to 41 °C) increased Prdx2 oxidation in erythrocytes. Oxygen binding is decreased when protons bind to hemoglobin (the Bohr effect), and lowering the oxygen affinity increases the rate of autoxidation of hemoglobin. The extent of Prdx2 oxidation can be thus a measure of oxidative stress to which erythrocytes are exposed; also in vivo, i.a., it can be a useful real-time marker of oxidative stress and an early indicator of sepsis [152]. The high level of Prdx2 in erythrocytes enables such an assay to be performed on a single drop of blood [154].

Prdx2 is not only a relevant antioxidant for the erythrocyte but also for the whole vasculature system. Indeed, erythrocytes have been shown to act as a sink for neutrophil-produced reactive oxidants [155]. About 55–60% of the plasma H_2_O_2_ ends up being consumed inside the erythrocyte, mainly by the erythrocyte Prdx2/Trx/TrxR system, whose efficiency, albeit low, was sufficient to reduce Prdx2 with a mean rate of 0.4 μM/s [46].

High-dose intravenous vitamin C infusions are used as an adjunctive or alternative cancer therapy. Exposure of erythrocytes to a high ascorbate concentration (20 mM) increased intracellular ascorbate concentration up to 1.5 mM and oxidized Prdx2; after 60 min, the oxidized dimer constituted 50–60% of the protein [156].

The reduction of oxidized Prdx2 was found to be significantly slower in older versus younger erythrocytes [157]. It has been speculated that oxidation to the disulfide can serve as a regulatory switch for other Prdx2-attributed roles such as stimulation of Ca^2+^-activated K^+^ transport or chaperone activity [158]. Surface accumulation of oxidized and oligomerized Prdx2 has also been postulated to serve as a means of physiological labeling oxidatively stressed erythrocytes [89].

Unexpectedly, Prdx2 was found to be resistant to hyperoxidation in the erythrocyte in comparison to Prdx2 in Jurkat cells. Treatment of erythrocytes (5 × 10^6^ cells/mL) with 5 μM of H_2_O_2_ completely converted Prdx2s into dimers without hyperoxidizing Prdx2 and oxidizing Hb [14]. It is unlikely that this effect was due to high levels of erythrocyte sulfiredoxin; in fact, this activity is low (k_cat_ = 0.2–0.5 min^−1^ at 30 °C) [63], and the reduction of hyperoxidized Prdx2 is especially slow in erythrocytes, likely because the Srx level in these cells is much lower than that in other cell types [159]. When hemolysate supplemented with Mg^2+^ and ATP was co-incubated with Jurkat lysates containing overoxidized Prdx, immunoblotting with antibodies to overoxidized Prdx showed that levels of hyperoxidation were not decreased. Rather, the slow turnover and continual persistence of the dimer limited the opportunity for the peroxidatic cysteines to become hyperoxidized. Thus, Prdx2 is able to act as an efficient scavenger of low concentrations of H_2_O_2_ without undergoing inactivation due to hyperoxidation [14]. No hyperoxidized forms of Prdx2 (as well as Prdx1 or Prdx6) were detected in old human erythrocytes (characterized by a higher density), indicating that they did not accumulate during the process of erythrocyte aging in vivo [157]. However, accumulation of hyperoxidized Prdx2 was found in old mouse erythrocytes [159].

It has been hypothesized that hyperoxidation does not occur easily in erythrocytes because it is unlikely that erythrocytes utilize the floodgate mechanism for signal transduction [14].

Nevertheless, more oxidizing conditions can cause hyperoxidation of Prdx2. When erythrocytes at a 50% hematocrit were incubated for 3 h with glucose oxidase at 0.1 mU/mL, which generated H_2_O_2_ at a rate of about 4.5 μM/min, ca. 10% of the Prdx2 was found to be hyperoxidized. The hyperoxidation was augmented by the inhibition of catalase, indicating that catalase protected Prdx2 against higher levels of H_2_O_2_ [160].

### 7.2. Erythrocyte Effects of Knockout of the Prdx2 Gene

No case of absence of Prdx2 in erythrocytes has been reported in humans so far, but knockout of the *Prdx2* has been performed in mice. The cells most affected in the *Prdx2*^−/−^ mice model were erythrocytes [91,161,162].

*Prdx2*^−/−^ knockout mice had splenomegaly and at 5 weeks had already developed a mild, chronic hemolytic anemia characterized by significantly decreased hematocrit and hemoglobin levels. Increased reticulocyte counts and erythropoietin levels were indicative of a compensatory effort to maintain hematologic homeostasis. Their erythrocytes contained more hemichromes than erythrocytes of wild-type mice (55.7 vs. 2.1 pmol/10^9^ cells) [162], contained Heinz bodies, and a variety of erythrocyte proteins were oxidized [161]. Erythrocytes of *Prdx2*^−/−^ mice released more microvesicles and showed a decreased elongation index (a measure of deformability) and accelerated senescence [99,162]. The survival half-time of erythrocytes of *Prdx2*^−/−^ mice was reduced (T_1/2_ of 6.8 d vs. 18.9 d for wild-type mice). Treatment of *Prdx2*^−/−^ mice with N-acetylcysteine (100 mg/kg for 3 weeks) prolonged erythrocyte survival in the circulation (T_1/2_ increased from 6.8 d to 8.9 d) [161,162]. Erythrocytes lacking Prdx2 also showed a greater increase in heme degradation during in vitro aging (incubation at 37 °C for >20 h) [163].

Erythrocytes from catalase knockout mice were very sensitive to exogenously applied hydrogen peroxide, which induces metHb formation. Data concerning erythrocytes lacking Prdx2 are divergent. Lee et al. noted increased MetHb content in erythrocytes from *Prdx2*^−/−^ mice treated with 200–500 μM of H_2_O_2_ [161], while Johnson et al. did not observe any increase in MetHb level in Prdx2-deficient erythrocytes treated with H_2_O_2_ in the same concentration range [164]. The only indicated difference was that Lee et al. incubated whole blood while Johnson et al. used purified, cellulose-filtered erythrocytes in Krebs-Ringer buffer with 10 mM of glucose. 

*Prdx2*^−/−^ mice showed constitutively increased intracellular hydrogen peroxide concentration [42,164]. The rate of irreversible catalase inhibition by 3-aminotriazole in erythrocytes is a measure of the rate of hydrogen peroxide production. On this basis, the rate of endogenous hydrogen peroxide production in wild-type mouse erythrocytes was estimated to be 23 nM/h. An increased rate of H_2_O_2_ generation (37 nM/h) was observed in *Prdx2*^−/−^ cells [163]. These results confirmed that Prdx2 is the main antioxidant controlling the intracellular H_2_O_2_ level.

### 7.3. Role of Prdx2 in the Disposal of Hydrogen Peroxide in Erythrocytes

What is the role of Prdx2 in the removal of H_2_O_2_ generated in erythrocytes and acting on these cells under physiological and pathophysiological conditions? Many results indicated that it is dominant under physiological conditions. Johnson et al. concluded from their model of erythrocyte H_2_O_2_ metabolism that deletion of Prdx2 increases the steady-state level of hydrogen peroxide in erythrocytes by about 3.9 times, deletion of GPx1 about 1.2 times, and deletion of catalase about 1.04 times [164]. In apparent agreement with this view, no erythrocyte abnormalities were found in human cases of acatalasemia [165] or in dogs, where the catalase activity is 30 times lower than in human erythrocytes [166,167].

Exposure of erythrocytes to higher H_2_O_2_ concentrations may dramatically change the contributions of H_2_O_2_-removing enzymes to the protection against H_2_O_2_-induced oxidative damage. For example, under H_2_O_2_ stress in vitro (10^9^ erythrocytes/mL exposed to 15–60 μM H_2_O_2_), inhibition of catalase induced a more significant decrease in the GSH/GSSG ratio, an increase in the membrane-bound hemoglobin, and an increase in the content of lipid peroxidation products in erythrocytes than inhibition of GPx or Prdx2 [168]. However, H_2_O_2_ concentrations used in vitro are usually higher than those occurring in vivo by several orders of magnitude, so they provide limited insight into in vivo situations.

### 7.4. Miscellaneous Antioxidant Effects in Erythrocytes

Prdx2 completely inhibited visible absorption spectral changes of oxyhemoglobin and the peroxidation of the erythrocyte membrane by a non-enzymatic Fe^3+^/O_2_/thiol mixed-function oxidase system [106].

Prdx2 is important for maintaining the proper level of *S*-nitrosohemoglobin (HbSNO), a mediator of the delivery of NO^●^ to smooth muscle cells. Inhibitors of Prdx2 2 and catalase significantly decreased erythrocyte HbSNO concentration. Prdx2 attenuates the stationary concentration of hydrogen peroxide and (indirectly) other oxidant species, which react with NO^●^, thereby decreasing its level and thus the level of HbSNO [169].

### 7.5. Role of Prdx2 in the Growth of Intracellular Parasites

Human Prdx2 is imported from the host erythrocyte into the cytosol of *Plasmodium falciparum* in the functional form and in significant concentrations, most likely to potentiate the own antioxidant defense of the parasite. Human Prdx2 accepts Trx1 of the parasite as a reducing substrate. Since *P. falciparum* is deficient in catalase and classical glutathione peroxidase, its redox balance relies on a complex set of peroxiredoxins [170]. The imported Prdx2 accounts for roughly half of the overall Trx-dependent peroxidase activity *P. falciparum.* The content of human Prdx2 in the parasite increases significantly after treatment with chloroquine, an agent that induces oxidative stress, suggesting that *P. falciparum* may be able to enhance the import of Prdx2 under oxidative pressure [171]. Parasite growth is prevented in red cells with inhibited Prdx2 [148,171]. Prdx2 as a potential therapeutic drug target has thus gained growing interest. Pretreatment of erythrocytes with conoidin I, a Prdx2 inhibitor, prevents the development of *Toxoplasma gondii* inside these cells [148]. Blocking uptake mechanisms of human proteins into the parasite might be another promising strategy for the development of future antimalarial drugs [171]. 

### 7.6. Circadian Oscillations of Prdx2 Hyperoxidation

The circadian rhythm is a widespread physiological phenomenon present in almost all organisms that is conditioned by a system of interlocked transcriptional/translational feedback loops. Oxidation–reduction cycles of Prdxs are a universal marker for circadian rhythms in all domains of life [172]. Interestingly, transcription-independent self-sustained oscillations of hyperoxidized Prdx2 with a period of about 24 h were detected in human erythrocytes, with the concentration of hyperoxidized Prdx2 peaking at noon and attaining the minimum at about midnight [173]. Saturating erythrocytes with CO abolished Prdx2 hyperoxidation, indicating that H_2_O_2_ produced from hemoglobin autoxidation is the main source of hyperoxidation of a small fraction of Prdx2. Circadian oscillation of the level of Prdx2–SO_2_H still occurred in mice lacking sulfiredoxin (*Srx*^−/−^). Purified 20S proteasome degraded Prdx2–SO_2_H preferentially over non-hyperoxidized Prdx2, so this proteasome seems to be responsible for the decay phase of Prdx2–SO_2_H oscillations and a gradual loss in Prdx2 during the life span of erythrocytes. About 1% of total Prdx2 was found to undergo daily oscillation in mouse erythrocytes. The band intensity for Prdx2–SO_2_H was found to increase with increasing density (and age) of the erythrocytes. Prdx1-deficient erythrocytes exhibited circadian oscillation of Prdx2–SO_2_H similar to that apparent for the wild-type cells. The amount of membrane-bound Prdxd2–SO_2_H oscillated in a phase opposite to that of total Prdx2–SO_2_H. These oscillations were hypothesized to be connected with the hyperoxidation-enhanced chaperone function of Prdx2 with respect to membrane proteins [159].

### 7.7. Chaperone Function

Prdx2 can have a dual function as an antioxidant enzyme or as a chaperone. Generally, molecular chaperones prevent protein aggregation by interacting with client proteins through unfolded and exposed areas. They only guide the transition of the client protein toward its folded form without being a component of the functional client. They may also help to restore misfolded proteins [174]. Prdx2 was found to act as a molecular chaperone in various cell models and cellular systems [41,78,104,158]. In model systems, human recombinant Prdx2 protected citrate synthase, malate dehydrogenase, and alcohol dehydrogenase against thermal aggregation and synuclein against ROS-induced aggregation [91,175,176]. Hyperoxidized Prdxs in association with Hsp70 formed complexes with misfolded proteins with the following recruitment of Hsp104. Then, Srx joined the complex and after reduction of the sulfinic acid, the complex disassociated, releasing the restored native protein [177]. In H_2_O_2_-treated Jurkat and human umbilical vein endothelial cells, the hyperoxidized Prdx2 selectively co-precipitated with the protein disulfide-isomerase ERp46. The resolving cysteine residue of Prdx2 was indispensable for the interaction to occur. The complex involved a stable non-covalent interaction that was disassociated by the reduction of intramolecular disulfides in ERp46 or by disruption of the decameric structure of hyperoxidized Prdx2. Reduced ERp46 reduced the dimeric form of Prdx2. In this respect, ERp46 acted as a substitute for Trx. Prdx2 can interact directly with other proteins with thioredoxin domains [54].

It has been suggested that in erythrocytes, Prdx2 may exert a chaperone function with respect to Hb and membrane proteins. In this context, the paper by Stuhlmeyer et al. [178] is sometimes cited due to misunderstanding. These authors reported that Antioxidant Protein 2 (AOP2) binds to Hb, showing an even higher affinity for heme than for Hb. The conserved cysteine 47 was essential for AOP2-heme binding. AOP2 prevented spontaneous and ascorbic acid-induced metHb formation. Binding of AOP2 to Hb protected or restored the active form of hemoglobin. Heme is the prosthetic group of several proteins and enzymes, so it was suggested that AOP2 may protect these other proteins [178]. However, AOP2 is a synonym for Prdx6, a one-Cys peroxiredoxin, and not for Prdx2.

Nevertheless, similar observations have been made for Prdx2. Co-immunoprecipitation analysis of erythrocyte lysates demonstrated that Prdx2 can interact with the hemoglobin α, β, and γ subunits but not with the δ of Hb. Hemolysates from *Prdx2*^−/−^ mice were more prone to H_2_O_2_-induced aggregation and Heinz body formation. The addition of recombinant human Prdx2 (rhPrdx2) restored the aggregation level of *Prdx2*^−/−^ hemolysates to that of wild-type hemolysates [100,179]. Experiments with truncated recombinant Prdx2 suggested that the binding site of Prdx2 to Hb is located in the N-terminus. A C-terminal truncated human Prdx2 protein did not show chaperone activity in vitro [175]. Mostly rhPrdx2 with a molecular weight around 220 kDa was bound to Hb, suggesting that Prdx2 protects Hb against H_2_O_2_-induced aggregation mainly as a decamer. Hb was proposed to be stabilized by binding to the decameric structure of Prdx2 [162,180].

Prdx2 was found to prevent metHb aggregation, suggesting that it may act as a chaperone for the denatured metHb [96,179]. Hemoglobin oxidation leads to the formation of hemichrome, a denatured form of this protein, which is a precursor of Heinz bodies. Prx2 was also shown to exhibit chaperone-like activity by retarding the precipitation of preformed hemichromes. By restricting the membrane binding of hemichromes, Prdx2 can impede Band 3 protein clustering. This mechanism plus the observed chaperone activity of Prdx2 for oxidized hemoglobin may help protect against hemolytic anemia [96].

It has been proposed that the chaperone function of Prdx2 is especially important in the stages of erythropoiesis and takes part in iron homeostasis as a chaperone for the synthesized Hb [162,164]. 

Membrane proteins were proposed to be other targets for the chaperone activity of Prdx2. It was suggested that generally, reduced Prdx2 can interact with Hb, and the oxidized Prdx2 will bind to cell membranes through the Band 3 protein and other membrane proteins [181] (Section 3.4). Prdx2 might act as a chaperone-like protein protecting key membrane proteins such as Band 3 against oxidation. This action of Prdx2 is associated with activation of Syk, an Src-family-related tyrosine kinase, which initiates the kinase-dependent intracellular signaling pathway, ending with the release of erythroid microparticles. In response to oxidation, Syk translocates to the membrane and induces Band 3 clusterization, resulting in membrane rearrangement and microvesiculation to clear damaged proteins [162]. In response to oxidative stress, Prdx2 is phosphorylated; its Tyr193 is a specific target of Syk kinase. Phosphorylation increases Prdx2 activity and translocates the protein to the membrane. Syk inhibitors block Prdx2 phosphorylation and membrane translocation. Syk is activated in diamide-treated erythrocytes. The Band 3 protein, a membrane binding site for Prdx2, is also the main target of Syk. In response to oxidative stress and Syk activation, the Band 3 protein becomes highly phosphorylated and forms clusters favoring membrane vesiculation and erythrocyte removal [182]. Syk-induced Tyr phosphorylation of Prdx2 also contributes to Prdx2 translocation to the membrane of erythrocytes exposed to diamide [137]. In erythrocytes of *Prdx2*^−/−^ mice, there was an increased association of the Syk kinase with the membrane, phosphorylation of membrane proteins (especially Band 3), increased binding of HSP27 and HSP70, and increased phagocytosis of these erythrocytes by macrophages in vitro [176].

### 7.8. Effect of Blood Storage on Prdx 2

Upon blood storage under blood-banking conditions, reduction of oxidized Prdx2 is slowed, which apparently accounts for the gradual increase in the content of oxidized Prdx2 in erythrocytes [183,184]. Prdx2 was found to remain predominantly reduced during the first 3 weeks of storage, and then the oxidized form accumulated progressively. In contrast to fresh cells, oxidation was not reversed by incubation of stored blood with glucose [183]. In another study, the extent of Prdx2 oxidation was found to increase slightly during incubation at 4 °C for up to 6 weeks and much more during subsequent incubation at 37 °C [24]. Surprisingly, no differences in the Trx levels, NADPH concentrations, or TrxR activity were found between fresh and stored erythrocytes [185]. Storage of erythrocytes in a high-pH, low-chloride, and high-phosphate/bicarbonate buffer (EAS-76v6) largely prevented accumulation of oxidized Prdx2 for at least 6 weeks, and dihydrolipoic acid but not Rejuvesol, N-acetylcysteine (NAC), or α-lipoic acid was able to reverse or protect against Prdx2 oxidation [183]. In another study, the storage-induced Prdx2 oxidation remained unchanged or even augmented in the presence of NAC (2.5–25 mM) [186]. Prdx2 redox status could thus be used as a biomarker of the quality of stored erythrocytes [183]. Attenuated Prdx2 activity is one mechanism contributing to a more pro-oxidative environment in the vascular compartment and problems associated with transfusion of older erythrocytes [185]. 

Blood storage under blood-banking conditions (for 28 days) led to the disappearance of the 100 kDa aggregate as well as the transformation of the 440 kDa complex into a 480 kDa complex and binding of a fraction of this complex to the membrane. A time-dependent linkage of the Prdx2 dimer to the membrane ranging from day 14 to day 28 was observed [187]. The addition of 5 mM NAC was unable to significantly prevent the formation of membrane-attached Prdx2 [186]. The variability of Prdx2 binding to the RBC membrane at different times may suggest that each blood donor has a different susceptibility to oxidative stress due to individual antioxidant activity levels. Donors showing increased membrane binding of Prdx2 in fresh RBCs exhibited increased membrane lipid peroxidation during storage [187]. The level of membrane-bound fraction of Prdx2 estimated using a quantitative proteomics approach showed a negative correlation with the extent of hemolysis in stored erythrocytes subjected to post-collection manipulations and thus can be another quality marker of erythrocytes to be transfused [188,189].

## 8. Erythrocyte Prdx2 in Pathologies

No or little oxidized Prdx2 is normally found in freshly isolated erythrocytes if oxidation prior to analysis is prevented. However, oxidative stress accompanying many diseases causes an increase in the levels of oxidized and hyperoxidized Prdx2 in various pathologies.

### 8.1. G6PD Deficiency

The antioxidant activity of erythrocyte Pdrx2 requires NADPH as the terminal reductant; it is produced in erythrocytes mainly by glucose 6-phosphate dehydrogenase (G6PD). The syndrome of G6PD deficiency limits NADPH production via the pentose phosphate pathway and prevents efficient recycling of Prdx2 in erythrocytes under oxidative stress. In a study by Cheah et al., Prdx2 in freshly isolated blood from neonates with G6PD deficiency was predominantly reduced, but the median level of oxidation was significantly higher (8%) than in the control neonates (3%). The G6PD-deficient erythrocytes were severely compromised in their ability to recycle Prdx2 oxidized by exogenous hydrogen peroxide, with only a 27% and 4% reduction after 1 h treatment with 0.1 or 1 mM of H_2_O_2_, respectively, compared with a >97% reduction in the control erythrocytes. The accumulation of oxidized Prdx2 in oxidatively stressed erythrocytes with G6PD deficiency suggests that impaired antioxidant activity of Prdx2 could contribute to the hemolysis and other complications associated with the condition [190].

### 8.2. Sickle Cell Disease

No differences in basal Prdx2 oxidation were seen between control or sickle cell disease (SCD) erythrocytes by some authors [191], while others found an increased oxidation [192]. No differences in Prdx2 recycling between control and sickle cell disease erythrocytes were also observed after the addition of exogenous hydrogen peroxide to these cells [191]. The enzymatic activity of Prdx2 was increased by a factor of 3.6-fold in SCD red cells with respect to healthy erythrocytes, apparently due to phosphorylation by activated Syk kinase [137]. Recycling of Prdx2 in erythrocytes from pediatric SCD patients was not significantly changed compared to erythrocytes from controls [157]. 

An increased association of decameric Prdx2 with the membrane of dense sickle cells was observed in early studies [193] and ascribed to changes in pH and changes or the concentration of calcium [37] and other ions [39].

More recently, an increased membrane binding of both monomers and dimers of Prdx2 was found to be significantly higher in erythrocytes of SAD mice, a transgenic mouse model of sickle cell disease, than in wild-type mouse red cells under normoxia, suggesting protection against membrane oxidative damage characterizing sickle red cells by the chaperone action of Prdx2. In the presence of iron, which increases ROS production, Prdx2 membrane binding was more marked in sickle erythrocyte fractions than in normal cells. In sickle erythrocytes of SAD mice exposed to hypoxia, Prdx2 membrane binding was reduced, suggesting that either the more damaged erythrocytes were already removed from circulation or that a higher amount of Prdx2 was required in the erythrocyte cytoplasm [97]. 

In SCD mouse erythrocytes, membrane translocation of not only Tyr-phosphorylated Prdx2 but also of active Syk was observed. Syk activation favors Band 3 clusterization, allowing the release of erythroid microparticles and erythrocyte membrane rearrangement [137]. 

### 8.3. β-Thalassemia

In erythrocytes of a transgenic mouse model of β-thalassemia, an increased expression of Prdx2 was found, most likely as an adaptive response to elevated oxidative stress experienced by these cells. In spite of this increase in the total Prdx2, the binding of Prdx2 to the membrane was markedly reduced. These changes seemingly contribute to the accumulation of oxidative damage, which seems to be mainly caused by oxidation of Prdx2 and subsequent dissociation from the erythrocyte membrane [94]. The levels of Prdx2 and Srx were not changed in patients with β-thalassemia intermedia but increased in beta thalassemia major (BTM). Prdx2 was present in microvesicles released from β-thalassemic erythrocytes [182]. Hyperoxidation of Prdx2 was observed in BTM patients [194]. 

Resveratrol, which accelerates erythroid maturation and ameliorates anemia in beta-thalassemic mice, increased the amount of Prdx2 associated with the membrane in β-thalassemic mouse red cells, suggesting that resveratrol increases the red cell lifespan in association with a decrease in red cell membrane oxidative damage [195].

### 8.4. Hereditary Spherocytosis

The presence of membrane-associated Prdx2 was found in erythrocytes of 21 among 57 hereditary spherocytosis (HS) patients but not in healthy controls. The occurrence of membrane-bound Prdx2 was associated with higher levels of oxidative stress as reflected by significantly increased membrane-bound hemoglobin in the same HS patients. No relation was observed between the presence of membrane-bound Prdx2 and the clinical severity of the disease [96]. In a larger follow-up study, however, no correlation was revealed between the amounts of membrane-bound hemoglobin and membrane-bound Prdx2 in HS patients. The presence of both inactive oxidized dimer and active reduced monomer forms of Prdx2 was found in the membrane-bound fraction, indicating that the membrane-bound Prdx2 was still (at least partly) functional and may be involved in the protection against lipid peroxidation [196].

### 8.5. Hereditary Ovalocytosis

No significant upregulation of total Prdx2 expression was observed in a homozygous patient with Southeast Asian ovalocytosis, but the membrane binding of the protein was increased [197].

### 8.6. Acute Anemia

Acute anemia leads to an increase in the bone marrow Prdx2 level. Prdx2 is produced and secreted in a cell-density-dependent manner by K562 cells, at least partly in the exosomes. In mice with acute anemia, gene and protein expression of Prdx2 was significantly increased in the bone marrow 2–3 days after bleeding. Prdx2 is the candidate mediator of osteoclastogenesis induced by hematopoietic cells. The addition of the purified K562 exosomal fraction to osteoclast precursors promoted osteoclast formation [198].

### 8.7. Obstructive Sleep Apnea

Significantly higher levels of Prdx2 hyperoxidation were observed in patients with obstructive sleep apnea (OSA) compared to those in healthy subjects. A significant correlation was evident between the severity of OSA and the levels of Prdx2 hyperoxidation. It has been suggested that hyperoxidized Prdx2 is a promising diagnostic marker candidate for OSA [199]. There were also diurnal variations in the extent of hyperoxidation in the patients: in the morning, the monomeric/dimeric forms of Prdx2 were more hyperoxidized in OSA erythrocytes compared to evening samples. Six months of positive airway pressure treatment decreased this hyperoxidation and generated multimeric overoxidized forms, perhaps associated with chaperone/transduction signaling activity of Prdx2 [200].

### 8.8. Cerebral Hemorrhage

Prdx2 plays a dual role in the cerebral hemorrhage. This protein is expressed in the mouse brain, mainly in neurons of the cerebral cortex, piriform cortex, and hypothalamus. Expression of Prdx2 was found to be elevated after subarachnoid hemorrhage and to protect again brain injury. Inhibition of Prdx2 promoted neuronal apoptosis by increasing the hydrogen peroxide level [201]. However, Prdx2 was the second most abundant protein in the cerebrospinal fluid of subarachnoid hemorrhage and traumatic brain injury patients due to release from necrotic neurons [202]. Extracellular Prdx2 in the cerebrospinal fluid of subarachnoid hemorrhage patients can interact with microglial toll-like 4 receptors (TLR4), inducing apoptosis of neurons [203].

Intracerebral hemorrhage leads to hemolysis. Lysis of erythrocytes is associated with neuronal death, brain edema, and neurological deficits. Although hemoglobin/iron and thrombin contribute to brain injury and neuroinflammation, it was reported that Prdx2 released from the red blood cells plays a key role as an initiator of inflammation. Large amounts of Prdx2 released from erythrocytes after intracerebral hemorrhage lead to brain damage [204]. Intracaudate injections of Prdx2 alone caused brain swelling, neuronal degeneration, neutrophil infiltration, leakage of the blood–brain barrier, and neurological deficits. All these injuries were attenuated by co-injection of conoidin A or heat inactivation of Prdx2 [205]. The mechanism by which Prdx2 released from erythrocytes causes damage is most probably linked again to the activation of microglial TLR4 [201]. Upon activation of TLR4, the microglia release inflammation mediators such as IL-1β and TNF-α, which may ultimately lead to neuronal cell death. It was proposed that the levels of Prdx2 in the CSF and the ratio of Prdx2 in the cerebrospinal fluid and the blood within 3 days of onset can be used as a biomarker to detect the severity of the disease and the clinical status of subarachnoid hemorrhage patients [206].

### 8.9. Neurological Diseases

Decreased Prdx2 level in erythrocyte membranes was found in abdominal aortic aneurysm patients [207]. The level of hyperoxidized Prdx2 was elevated in erythrocytes of Alzheimer’s disease (AD) patients [208]. Moreover, nitrated Prdx2 was detected in the brains of patients with early AD [209]. An increased level of *S*-nitrosylated Prdx2 was observed in human Parkinson’s disease brains [77]. The level of membrane-bound Prdx2 was not changed in erythrocytes of patients with autism spectrum disorder, but the blood plasma level of Prdx2 was increased [210].

### 8.10. Endotoxemia

Exposure of human erythrocytes to neutrophils activated by the lipopolysaccharide of *Staphylococcus aureus* induced a rapid transient increase in Prdx2. In whole animal experiments, erythrocyte Prdx2 oxidation in endotoxemic mice (injected with lipopolysaccharide of *E. coli*) increased rapidly from 6 h to a maximum at 10 h, after which it gradually decreased over the next 24 h [152].

## 9. Concluding Remarks

Prdx2 belongs to the main antioxidants that protect erythrocytes under physiological conditions against H_2_O_2_, other peroxides, and peroxynitrite. It reacts quickly with hydrogen peroxide (k > 10^7^ M^−1^ s^−1^). Prdx2 is the main protein that disposes of low levels of hydrogen peroxide produced by hemoglobin autoxidation, but higher doses of hydrogen peroxide transiently inactivate it because its reduction (mainly by thioredoxin/thioredoxin reductase) is relatively slow. Apparently, oxidized Prdx2 may be also reduced in situ by glutathione and other thiols. Prdx2 is subject to several post-translational modifications, some of which (phosphorylation, nitration, and acetylation) may increase its activity. Erythrocytes of *Prdx2^−/−^* mice have a shortened lifespan and increased protein oxidation. Circadian oscillations in the level of Prdx2 were reported. The intraerythrocyte parasite *Plasmadium falciparum* imports the host’s Prdx2 for its own defense. Prdx2 may play a chaperone role with respect to hemoglobin and membrane proteins. The extent of oxidation, hyperoxidation, and membrane binding of Prdx2 may serve as markers of oxidative stress, which is useful in the assessment of the severity of various diseases. The latter is complicated by the lack of a simple method of estimation of its activity, oxidation, and hyperoxidation (electrophoresis and blotting are time- and reagent-consuming), but the development of advanced analytical methods may change this situation, at least for well-equipped laboratory centers.

## Figures and Tables

**Table 1:** Selected rate constants of Prdx2 reactions (data for a C172S mutant). After [55].

Reaction	Rate Constant
Prdx-SH + H_2_O_2_ → Prdx-SOH	2.7 × 10^7^ M^−1^ s^−1^
Prdx-SOH + H_2_O_2_ → Prdx-SO_2_H	12,000 M^−1^ s^−1^
Prdx-SOH → Prdx(SS)	2 s^−1^
Prdx-SOH + GSH → Prdx-SSG	500 M^−1^ s^−1^
